# Unusual cause of generalized osteolytic vertebral lesions: a case report

**DOI:** 10.1186/1752-1947-1-33

**Published:** 2007-06-26

**Authors:** Sudip Nanda, Surya Prakash Bhatt, David Steinberg, Stephen A Volk

**Affiliations:** 1 Department of Internal Medicine, St. Luke's Hospital, 801 Ostrum Street, Bethlehem, Pennsylvania 18015, USA; 2 Department of Pathology, St. Luke's Hospital, 801 Ostrum Street, Bethlehem, Pennsylvania 18015, USA; 3 Department of Hematology and Oncology, St. Luke's Hospital, 801 Ostrum Street, Bethlehem, Pennsylvania 18015, USA

## Abstract

**Background:**

Vertebral sarcoidosis is an extremely rare form of osseous sarcoidosis. Although osseous sarcoidosis is almost always an incidental finding of sarcoidosis elsewhere in the body, vertebrae may be the primary disease site. Involvement of vertebrae is usually localized and sclerotic or lytic.

**Case presentation:**

We describe a case of extensive asymptomatic vertebral involvement by sarcoid with osteolytic lesions. Making the diagnosis requires biopsy and ruling out other commoner causes of osteolytic vertebral lesions.

**Conclusion:**

We report this case in the hope of expanding the knowledge of osseous sarcoidosis. Our patient was unique in that all involvement was axial with sparing of the peripheral skeleton, near absence of any other organ involvement, diffuse involvement of the whole spine and osteolytic bone lesions.

## Background

Vertebral sarcoidosis is increasingly diagnosed secondary to the recent advances in imaging studies. Diagnosis is made by a combination of clinical, radiographic and histologic findings. Histopathology is the cornerstone in ruling out differentials like tuberculosis, Langerhans' cell histiocytosis and tumour related sarcoid reactions. We report extensive, asymptomatic, axial, osteolytic vertebral lesions with absence of any significant involvement of any other organ systems.

## Case presentation

A 44 year old white female with diabetes presented with symptoms and signs suggestive of acute appendicitis. Review of systems was negative for fever, cough, dyspnea, chest pain, joint pains, change in bowel habits and weight loss. Physical examination was normal except for tenderness in the right iliac fossa without guarding or rebound. The white cell count was 9130/μL. Liver and renal function tests were normal. CT of the abdomen revealed fatty liver, changes of acute appendicitis and an incidental finding of multiple lytic lesions in the vertebral bodies. She underwent appendectomy and recovered uneventfully.

**Figure 1:**
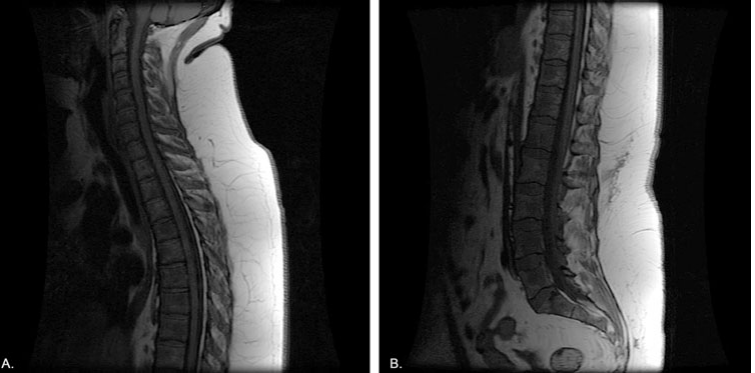
**MRI showing (A) cervico-thoracic involvement and (B) thoraco-lumbar involvement**. T2 weighted image highlighting heterogenous involvement of the entire spine with preserved disk space and absence of fractures.

**Figure 2:**
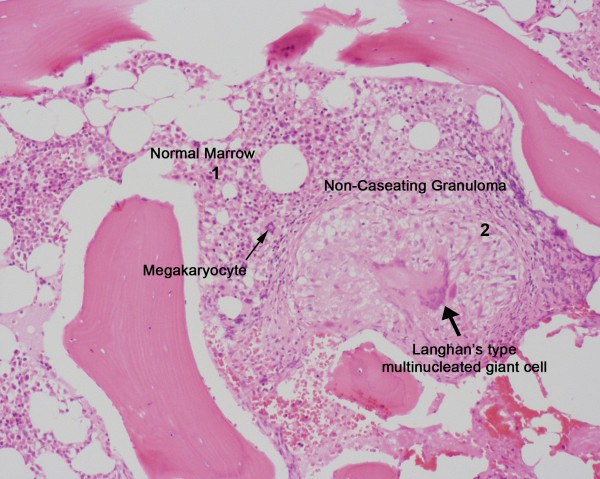
**Non-caseating granuloma**. Hematoxylin and Eosin stain. (× 200). Normal trabecular bone and trilineage bone marrow (1) showing megakaryocytes (thin arrow). A discrete well-formed non-caseating granuloma (2) composed of histiocytes, epitheloid cells and Langhan's type multinucleated giant cell (thick arrow).

**Figure 3:**
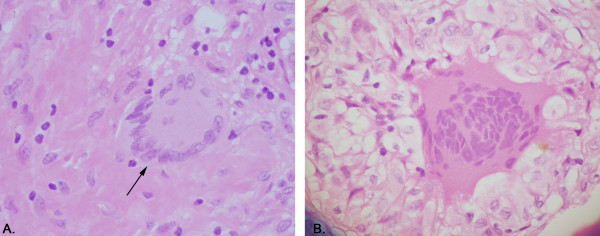
**(A) Langhan's giant cell and (B) Foreign body giant cell**. Hematoxylin and Eosin stain. (× 400). Photomicrograph of the giant cell with surrounding histiocytes and occasional lymphocytes.

A follow up CT scan of spine and chest revealed small lucent lesions throughout the spine and bilateral lower lobe non-calcified pulmonary nodules. Purified protein derivative (PPD) skin testing for tuberculosis was negative. MRI of the spine revealed diffuse bone marrow heterogeneity throughout the cervico-thoracic and thoraco-lumbarspine (Figure 1A and 1B). Myeloma, lymphoma, and metastatic disease were considered amongst the differential diagnoses. Serum electrophoresis revealed low total protein and albumin, normal immunoglobulins and no monoclonal bands. Needle biopsy of the fourth lumbar vertebra was unremarkable. Immunostains were negative for malignancy. Bone marrow aspiration and biopsy revealed non-caseating epithelioid cell granulomas in the background of normal hemopoietic cells (Figures 2 and 3). Bone marrow had normal karyotype. Flow cytometry did not reveal any monoclonal B-cells or abnormal T-cells. Cultures for bacteria, fungi and acid fast bacilli were negative. The angiotensin converting enzyme assay was normal.

## Discussion

The patient was diagnosed with sarcoidosis. The other differentials of generalized osteolytic vertebral lesions which included tuberculosis, metastasis, multiple myeloma, lymphoma, eosinophilic granuloma and disseminated echinococcal lesion were ruled out by biopsy and investigations. To the best of our knowledge this represents the most extensive reported case of asymptomatic vertebral involvement by sarcoidosis involving cervical, thoracic and lumbar regions.

The patient did not have a history of tuberculosis or exposure to the same. Skin anergy is typical in patients with sarcoidosis but they react very strongly to PPD when they develop active tuberculosis [1]. Histopathology did not reveal any caseating granuloma and bone marrow was negative for acid fast bacilli and fungus on culture. Though axial tuberculosis may be culture negative, this patient's predisposition, presentation, PPD test and histopathology were not consistent with tuberculosis.

Well defined non-caseating granulomas effectively ruled out Langerhans'-cell histiocytosis. The unifying feature of the group of diseases designated as Langerhans'-cell histiocytosis is infiltration by Langerhans' cell. Instead of the well formed granuloma seen in sarcoidosis, there is a polymorphic appearance from an admixture of Langerhans' cell, nonspecific histiocyte, lymphocytes and eosinophils. Langerhans' cell also has a characteristic morphologic appearance on high power with the nuclei often being lobulated or indented with a longitudinal groove [2]. If preliminary histopathology is suggestive, histochemical studies like staining for S-100 protein, CD1a and HLA-DR are done to confirm Langerhans' cell. However the mere presence of Langerhans' cell is not diagnostic of Langerhans'-cell histiocytosis [3]. A specific intracytoplasmic organelle, known as Langerhans' or Birbeck granule seen on electron microscopy can confirm the diagnosis.

Involvement of bone occurs in 5% of patients with sarcoidosis. Although bony involvement may be the earliest manifestation, it usually accompanies skin lesions [4]. The first major review of osseous sarcoidosis by Neville et al [5] noted bone involvement in 24 patients in whom hands and feet were always involved and involvement elsewhere was seen in four of them. Soft tissue involvement was present in half of the patients. Bone involvement was usually an incidental finding. In this review, patients usually presented with lung involvement (75%), lupus pernio (50%), chronic skin lesions (41%), eye (51%), lymph nodes (21%), liver (17%), spleen (13%), parotids (13%) and facial palsy in two patients. Lungs were the most common site of disease.

There are three distinct types of bone lesions – 1) lytic cortical defects which are usually rounded and heal forming punched out cysts 2) permeative defects which show progressive cortical tunneling with remodeling of trabecular and cortical architecture and 3) destructive lesions with a secondary joint surface involvement and periosteal reaction. Bone lesions imply a chronic severe disorder. Usually proximal and middle phalanges are involved. However skull, vertebra, ribs, maxilla and nasal bones may be affected. Cystic bone lesions are the predominant variant. A very small number have spine lesions [6]. When involved, lower thoracic and upper lumbar vertebrae are usual sites although cervical spine may also be involved [7,8]. Axial involvement which may be sclerotic or osteolytic, is usually localized to a region of the vertebral column and is mostly symptomatic. A series of three patients with vertebral sarcoidosis where all had pain and two had vertebrae as the primary site affected has been reported [9]. In involvement of the spine, disk spaces are usually preserved. As imaging studies have become common, more people have detectable skeletal involvement with osteoporosis and osteopenia.

Radiologically cystic, lacelike honeycomb or extensive bone erosions, when accompanied by intact articular space and accompanied by soft tissue mass or tenosynovitis is virtually diagnostic of sarcoidosis. CT scan reveals osteolytic or osteosclerotic lesions. MRI in vertebral sarcoidosis may show varied T1 weighted, T2 weighted and STIR sequence images depending on nature (osteolytic vs. osteosclerotic) and activity (active inflammation vs. healed) of lesions [9]. FDG positron emission tomography is an investigation whose role in skeletal sarcoidosis is still being defined. Its ability to distinguish sarcoidosis from malignant conditions has been suggested but this needs to be verified by other observers [10]. However none of the imaging finding can be considered diagnostic and biopsy is required to confirm the diagnosis.

Before making a diagnosis of sarcoidosis, all other causes of non-caseating epithelioid granuloma must be eliminated. Non-caseating granulomas are seen in 4% of regional lymph nodes with carcinoma, 14% of patients with Hodgkin's disease, 7% of patients with non-Hodgkin's lymphoma, 7% of patients with primary seminoma and dysgerminoma [11]. Tumor related granulomas are so similar to sarcoid granulomas that they are often called tumor-related sarcoid reactions. Reported as early as 1911, they have been studied extensively [12]. They can occur within the primary tumor, draining lymph nodes or at distant sites like the spleen and bone-marrow. Pathogenesis remains elusive and they may be manifestations of host response to tumor antigens. Brincker proposed a way to differentiate them based on number of B lymphocytes in the central part of the granuloma [13]. According to this original report, sarcoid granulomas were devoid of central B cells while tumor related sarcoid reactions had B-cells in the centre [11,13]. Subsequently it was found that this distinction is not absolute and Brincker reported B-cell negative granulomas in tumor associated sarcoid reactions. B cell negative sarcoid reactions have subsequently been reported in melanoma [14] and other cancers. The term atypical tumor-related sarcoid granuloma has been coined for these malignancy related granulomas that are B-cell deficient. Thus while tumor related granulomas can be B-cell positive or negative, those seen in sarcoidosis are predominantly associated with T-cells. The marrow granuloma in our case was positive for T cells (CD45, CD3, CD4, and CD8), histiocytes (CD68) and few plasma cells (CD138). The marrow histopathology and culture was negative for infectious causes of granuloma like tuberculosis and fungus which are also T cell granulomas. There was no evidence of this being a hypersensitivity reaction or any exposure to beryllium, titanium or aluminum.

To summarize, vertebral sarcoidosis although extremely rare is being increasingly diagnosed with advances in imaging; though osseous sarcoidosis is an incidental finding of sarcoidosis elsewhere in the body, vertebrae may be the primary disease site; vertebral involvement is usually localized but may be generalized as in our patient; vertebral sarcoidosis is mostly focal osteolytic or combined – lytic and sclerotic, and rarely generalized osteolytic as seen here; and while usually symptomatic, vertebral involvement may be totally asymptomatic. Making the diagnosis requires biopsy and analysis to rule out infections, malignancy and other causes. We describe a unique case of vertebral sarcoidosis with no symptoms from bone involvement, no obvious presence of sarcoidosis in other organs except for incidental pulmonary nodules, and perhaps the most extensive asymptomatic involvement of the axial skeleton.

Vertebral sarcoidosis is so rare that there are no guidelines for its treatment. Therapy includes corticosteroids, methotrexate and other disease modifying agents for control of disease activity; bisphosphonates, calcium and vitamin D for treatment of osteoporosis; and vertebroplasty for fracture. Although initially the patient preferred not to be treated being asymptomatic, she developed backache four months later and is currently on non-steroidal anti-inflammatory drug (NSAID), bisphosphonates and steroid therapy.

## Conclusion

We report this case in the hope of expanding the knowledge of osseous sarcoidosis. Skeletal sarcoidosis is usually peripheral, involves the axial skeleton secondary to other organ involvement, is often localized to a few vertebrae and is combined – osteosclerotic and osteolytic. Our patient was unique in that all involvement was axial with sparing of the peripheral skeleton and had near absence of any other organ involvement, diffuse involvement of the whole spine and osteolytic nature of bone lesions.

## Abbreviations

CT-computed tomography, MRI-magnetic resonance imaging, PPD-purified protein derivative, NSAID-non-steroidal anti-inflammatory drug, FDG PET-Fluoro-deoxy-glucose Positron Emission Tomography

## Competing interests

The author(s) declare that they have no competing interests.

## Authors' contributions

SN has been involved in the conception, design, drafting and revising the manuscript. SPB has revised and critically analyzed the manuscript for important intellectual content. DS has been involved with the pathological diagnosis of the patient and revision of manuscript. SAV has been involved in the diagnosis and treatment of the patient and in revising the manuscript
